# Lack of strategic service provisioning by Pederson’s cleaner shrimp (*Ancylomenes pedersoni*) highlights independent evolution of cleaning behaviors between ocean basins

**DOI:** 10.1038/s41598-018-37418-5

**Published:** 2019-01-24

**Authors:** Benjamin M. Titus, Marymegan Daly, Clayton Vondriska, Ian Hamilton, Dan A. Exton

**Affiliations:** 1 Department of Evolution, Ecology, and Organismal Biology, The Ohio State University, 1315 Kinnear Rd, Columbus, Ohio 43210 USA; 2 Operation Wallacea, Wallace House, Old Bolingbroke, Spilsby, Lincolnshire PE23 4EX UK; 3 Division of Invertebrate Zoology, The American Museum of Natural History, New York, NY 10024 USA; 4 Department of Biological Sciences, Arkansas State University, Jonesboro, AR 74267 USA

## Abstract

Marine cleaning interactions have been useful model systems for exploring evolutionary game theory and explaining the stability of mutualism. In the Indo-Pacific, cleaner organisms will occasionally “cheat” and remove live tissue, clients use partner control mechanisms to maintain cleaner honesty, and cleaners strategically increase service quality for predatory clients that can “punish” more severely. The extent to which reef communities in the Caribbean have evolved similar strategies for maintaining the stability of these symbioses is less clear. Here we study the strategic service provisioning in Pederson’s cleaner shrimp (*Ancylomenes pedersoni*) on Caribbean coral reefs. In the Gulf of Honduras, we use video observations to analyze >1000 cleaning interactions and record >850 incidents of cheating. We demonstrate that *A. pedersoni* cheat frequently and do not vary their service quality based on client trophic position or cleaner shrimp group size. As a direct analog to the cleaner shrimp *A. longicarpus* in the Indo-Pacific, our study highlights that although cleaning interactions in both ocean basins are ecologically analogous and result in parasite removal, the strategic behaviors that mediate these interactions have evolved independently in cleaner shrimps.

## Introduction

The evolution and stability of intraspecific cooperation and interspecific mutualism (terms we use as defined by^1–4^) have long fascinated evolutionary biologists, and are generally explained by one of two classic scenarios: (i) helping is the by-product of a self-serving act, or (ii) there is concerted investment by both partners that yields future fitness benefits (reviewed by^4^). The latter creates an inherent incentive to cheat, or defect, as the immediate benefits of cheating outweigh the immediate benefits of investing. Under certain game theoretical models (e.g. the iterated prisoner’s dilemma), selection should drive the evolution of partner control mechanisms to regulate interactions, at which point helping behaviors emerge as stable evolutionary outcomes^2,3,5,6^.

In some settings (e.g. symbioses), large intraspecific groups provide services simultaneously to the same client as part of a larger interspecific mutualism; a situation analogous to the iterated prisoner’s dilemma. Here, complex decisions may be required by each individual regarding the provision of honest and dishonest behaviors, and their standing in the larger intraspecific group and interspecific interactions^4,6–8^. Bshary *et al*.^6^ developed a model that predicts two stable evolutionary strategies for this scenario: (i) service providers should cheat immediately to gain maximal fitness benefits leading to a complete breakdown in service quality, or (ii) service quality should be greater when provided in pairs, in an iterated game, than by single service providers. These predictions rely on the assumption that each client mediates the interaction in a similar manner (i.e. cheating is always met with the same level of punishment). Variation in a client’s ability to punish defectors may lead to scenarios where service providers, either singly or in pairs, may strategically alter service quality based on perceived risk of expulsion from an intraspecific social group, alongside the severity of interspecific punishment. Under these scenarios, clients with the ability to punish cheating more severely would be predicted to receive high quality services from both single and paired providers, while greater variation in service quality should manifest itself in clients that are unable to punish as severely^7,9,10^.

In marine systems cleaning symbioses have been classic model systems for investigating game theory and the stability of intra- and inter-specific cooperation and mutualism^6–9,11^. Cleaning interactions are predicated on mutualistic behaviors between one or more cleaners (the service providers) and client reef fish. Cleaners, typically fish or shrimps, remove potentially harmful ectoparasites from clients that pose motionless at cleaning stations, and clients do not prey on small, vulnerable cleaners. Cleaning is done singly or in intraspecific groups and cleaner presence has been shown to positively impact fish health and diversity on coral reefs^12–16^. However, not every cleaning interaction is mutualistic. Some cleaner organisms have shown preference for nutritive sources other than client ectoparasites such as fish mucus or live tissue, and thus, an inherent incentive to cheat exists for some cleaners^17^. Partner control mechanisms (e.g. partner switching, aggressive chasing) imposed by the client on the cleaners have evolved to mediate incidences of cheating, and cleaner service quality varies depending on cleaner group size and client trophic position^9,10,18^.

In the tropical Indo-Pacific, both cleaner wrasse (*Labroides dimidiatus*) and cleaner shrimp (*Ancylomenes longicarpus*) have been shown to provide greater service quality to predatory than herbivorous clients^6,10^. Cleaning in intraspecific groups of two or more has been shown to reduce incidences of cheating in cleaner wrasse, and while its role remains less clear for cleaner shrimp, larger group sizes have shown a reduction in cheating^6,10^. Partner control mechanisms appear to be well developed on Indo-Pacific reefs, and antagonistic displays of aggression by clients have been documented towards both cleaner types^6,10^. However, not all cleaning symbioses may so clearly meet the expectations established by game theory. Caribbean cleaner gobies (genus *Elacatinus*) appear to show no strategic adjustment of service quality based on client trophic position, and the symbiosis appears to be a system without punishment or partner control^8,19–21^. At most, clients leave immediately after a cheating incident, but no physical punishment has been documented. Some evidence exists that male cleaner gobies reduce cheating when cleaning in intraspecific pairs, but service quality remains high regardless of group size, in contrast to Indo-Pacific cleaner wrasse^8^. Whether cleaner gobies are directly comparable to cleaner wrasses and broadly representative of how stable cleaning strategies have evolved in both ocean basins is unclear. Wrasses and gobies are not taxonomic analogs and there are notable ecological differences between cleaner types^17,21^. Further, there is a lack of research on strategic service behaviors from non-fish cleaners in the Caribbean. This perspective is needed to broaden the taxonomic scope of the cleaning literature and begin to synthesize behavioral patterns that have evolved across ocean basins.

On Caribbean coral reefs, cleaner shrimps are important cleaners, and although they have been less studied than cleaner fishes, recent research has shown that they are visited frequently, have broadly overlapping client diversity with cleaner gobies, and are effective at reducing parasite loads on reef fishes^22–28^. Among the most well studied and ecologically important Caribbean cleaner shrimps is Pederson’s cleaner shrimp, *Ancylomenes pedersoni*; a dedicated cleaner (i.e. a species that cleans for all of its non-larval ontogeny)^29^ that associates with sea anemones and that cleans over 20 families of client reef fishes^23–26^. Whereas behavioral comparisons between Caribbean cleaner gobies *Elacatinus* spp. and the Indo-Pacific cleaner wrasse *L. dimidiatus* are not direct analogs, as they do not share a recent common ancestor and are known to prefer different food sources (i.e. fish mucus vs. ectoparasites), *A. pedersoni* is a direct analog to *A. longicarpus*. They belong to the same genus, are both symbiotic with sea anemones, perform cleaning services singly or with multiple conspecifics, and both are known to cheat client reef fish^10,23,27,30,31^. Here we investigate the effect of cleaner group size and client trophic position on strategic service provisioning of *A. pedersoni* in the Gulf of Honduras to fill an important gap in our knowledge on the evolution of cleaning behaviors on tropical coral reefs. Namely, we aim to provide critical data to explore whether strategic cleaning behaviors have converged on similar or dissimilar strategies in separate ocean basins. With these data, we ask whether *A. pedersoni*, like *A. longicarpus*, strategically adjusts service quality based on client trophic position and intraspecific group size, or whether, like the co-occurring cleaner gobies in the Caribbean, there is no adjustment of service quality for any client or group size.

## Results

We recorded a total of 316 hours of video footage from 23, 17, and 18 cleaning stations at Utila, Cayos Cochinos, and Banco Capiro respectively. Shrimp group sizes ranged from 1–12 individuals at Utila (median = 2, IQR = 1–2), and 1–11 at both Cayos Cochinos (median = 3, IQR = 2–3.7) and Banco Capiro (median = 4, IQR = 1.75–6.25). A total of 1028 *A. pedersoni*-only cleaning interactions were recorded across all reef sites: Utila = 344, Cayos Cochinos = 349, and Banco Capiro = 335.

### Client diversity

We recorded cleaning interactions for 16 families, 24 genera, and 48 species (Table 1). Per site we recorded 26, 27, and 22 client species at Utila, Cayos Cochinos, and Banco Capiro respectively. Of these, 28 client species were categorized as predatory and 20 client species were non-predatory (Table 1). While some common patterns in client diversity are apparent, e.g. parrotfishes were commonly cleaned clients across all sites, the frequency at which different species visited cleaning stations at each site varied considerably. At Utila, longfin damselfish (*Stegastes diencaeus*) and schoolmaster snapper (*Lutjanus apodus*) represented >50% of all observed cleaning interactions (Table 1). At Cayos Cochinos, grasby groupers (*Cephalopholis cruentata*) and redband parrotfish (*Sparisoma aurofrenatum*) represented >40% of all cleaning interactions, and at Banco Capiro, four parrotfish species represented >55% of all observed cleaning interactions (Table 1).

**Table 1:** Summary of client reef fish diversity observed at *Ancylomenes pedersoni* cleaning stations in this study, with raw number of observations for that species at each reef site (Banco Caprio, Cayos Cochinos, and Utila), and the percent of included cleans at that site featuring that species.

Family	Genus	Species	Common Name	Predatory?	Banco Capiro # (%)	Cayos Cochinos # (%)	Utila # (%)
Acanthurdiae	*Acanthurus*	*bahianus*	Ocean Surgeonfish	No	53 (16%)*	1 (<1%)	0 (0%)
		*chirurgus*	Doctorfish	No	1 (<1%)	14 (4%)	4 (1%)
		*coeruleus*	Blue Tang	No	3 (<1%)	3 (<1%)	15 (4%)
Chaetodontidae	*Chaetodon*	*capistratus*	Foureye Butterflyfish	No	2 (<1%)	1 (<1%)	3 (<1%)
		*oceallatus*	Spotfin Butterflyfish	No	4 (1%)	0 (0%)	0 (0%)
		*striatus*	Banded Butterflyfish	No	3 (<1%)	0 (0%)	0 (0%)
Haemulidae	*Haemulon*	*aurolineatum*	Tomtate	Yes	5 (2%)	0 (0%)	0 (0%)
		*macrostomum*	Spanish Grunt	Yes	0 (0%)	2 (<1%)	0 (0%)
		*plumierii*	White Grunt	Yes	20 (6%)	33 (9%)*	5 (2%)
		*sciurus*	Bluestriped Grunt	Yes	0 (0%)	20 (6%)*	0 (0%)
Holocentridae	*Holocentrus*	*rufus*	Longspine Squirrelfish	Yes	6 (2%)	0 (0%)	2 (<1%)
Labridae	*Halichoeres*	*bivittatus*	Slippery Dick	Yes	0 (0%)	0 (0%)	1 (<1%)
		*garnoti*	Yellowhead Wrasse	Yes	4 (1%)	0 (0%)	5 (2%)
Lutjanidae	*Lutjanus*	*apodus*	Schoolmaster	Yes	0 (0%)	0 (0%)	69 (20%)*
		*cyanopterus*	Cubera Snapper	Yes	0 (0%)	2 (<1%)	0 (0%)
		*griseus*	Gray Snapper	Yes	0 (0%)	14 (4%)	0 (0%)
		*mahogoni*	Mahogany Snapper	Yes	0 (0%)	0 (0%)	1 (<1%)
Monacanthidae	*Cantherhines*	*pullus*	Orange-Spotted Filefish	No	18 (5%)	0 (0%)	0 (0%)
Mullidae	*Mulloidichthys*	*martinicus*	Yellowtail Goatfish	Yes	0 (0%)	8 (2%)	0 (0%)
	*Pseudupeneus*	*maculatus*	Spotted Goatfish	Yes	0 (0%)	0 (0%)	1 (<1%)
Ostraciidae	*Lactophrys*	*bicaudalis*	Spotted Trunkfish	Yes	0 (0%)	1 (<1%)	0 (0%)
		*trigonus*	Buffalo Trunkfish	Yes	0 (0%)	1 (<1%)	0 (0%)
		*triqueter*	Smooth Trunkfish	Yes	1 (<1%)	0 (0%)	0 (0%)
Pomacentridae	*Chromis*	*cyanea*	Blue Chromis	No	0 (0%)	16 (5%)	6 (2%)
	*Pomacanthus*	*arcuatus*	Gray Angelfish	No	0 (0%)	12 (3%)	6 (2%)
	*Stegastes*	*diencaeus*	Longfin Damselfish	No	0 (0%)	0 (0%)	105 (31%)*
		*partitus*	Bicolor Damselfish	No	0 (0%)	23 (7%)*	2 (<1%)
		*variabilis*	Cocoa Damselfish	No	2 (<1%)	0 (0%)	0 (0%)
Scaridae	*Sparisoma*	*aurofrenatum*	Redband Parrotfish	No	58 (17%)*	70 (20%)*	18 (5%)^
		*chrysopterum*	Redtail Parrotfish	No	0 (0%)	1 (<1%)	0 (0%)
		*radians*	Bucktooth Parrotfish	No	0 (0%)	1 (<1%)	0 (0%)
		*viride*	Stoplight Parrotfish	No	35 (10%)*	5 (1%)	7 (2%)
	*Scarus*	*guacamaia*	Rainbow Parrotfish	No	0 (0%)	0 (0%)	4 (1%)
		*hypselopterus*	Yellow-tail Parrotfish	No	0 (0%)	3 (<1%)	0 (0%)
		*iseri*	Striped Parrotfish	No	41 (12%)*	11 (3%)	10 (3%)
		*taeniopterus*	Princess Parrotfish	No	57 (17%)*	10 (3%)	24 (7%)*
Serranidae	*Cephalopholis*	*cruentata*	Graysby	Yes	9 (3%)	76 (22%)*	18 (5%)^
	*Epinephelus*	*guttatus*	Red Hind	Yes	0 (0%)	0 (0%)	2 (<1%)
		*striatus*	Nassau Grouper	Yes	0 (0%)	0 (0%)	1 (<1%)
	*Mycteroperca*	*tigris*	Tiger Grouper	Yes	0 (0%)	0 (0%)	7 (2%)
	*Serranus*	*tigrinus*	Harlequin Bass	Yes	3 (<1%)	0 (0%)	0 (0%)
Sparidae	*Calamus*	*calamus*	Saucereye Porgy	Yes	0 (0%)	1 (<1%)	0 (0%)
		*Nodosus*	Knobbed Porgy	Yes	0% (0%)	0 (0%)	1 (<1%)
		*pennatula*	Pluma Porgy	Yes	1 (<1%)	0 (0%)	0 (0%)
Synodontidae	*Synodus*	*foetens*	Inshore Lizardfish	Yes	0 (0%)	5 (1%)	2 (<1%)
Tetraodontidae	*Canthigaster*	*rostrate*	Sharpnose Puffer	Yes	8 (2%)	14 (4%)	24 (7%)*
	*Sphoeroides*	*spengleri*	Bandtail Puffer	Yes	0 (0%)	1 (<1%)	0 (0%)
Urotrygonidae	*Urobatis*	*jamaicensis*	Yellow Stingray	Yes	1 (<1%)	0 (0%)	0 (0%)

Clients were identified to species and their trophic position (Predatory? Yes/No) recorded. * denotes the five most frequently cleaned client species at that reef site.

### Behavioral observations

Across 1028 cleaning interactions, 619 were conducted by solitary shrimp (~60%) and 409 were conducted in groups of 2 or more (~40%). 657 cleans were recorded of non-predatory clients and 371 were recorded of predatory clients. We observed a total of 855 individual incidents of cheating (i.e. jolts or flinches), with a median cheat rate min^−1^ of 0.0 (IQR = 0.0–2.89 cheats min^−1^). 614 cleaning interactions showed no evidence of cheating (~60%), and 414 cleaning interactions showed evidence of at least one cheating occurrence (~40%). Of those interactions showing no incidents of cheating, 381 were conducted by solitary shrimp (62%) while the remaining 233 were conducted in groups (38%). Across the 414 cleaning interactions where cheating occurred, 312 were conducted by a solitary shrimp (~75%) and 102 were conducted by groups (25%). Where cheating was observed, a single cheating event was observed in 249 interactions (60%). In 165 interactions (40%) we observed multiple incidents of cheating. The maximum number of cheats observed in a single interaction was 56, between a group of cleaner shrimp and a lizardfish (*Synodus foetens*), during an interaction lasting >13 min. No antagonistic displays of aggression were observed by any client towards cleaner shrimp following cheating or at any other time during recordings.

**Figure 1:**
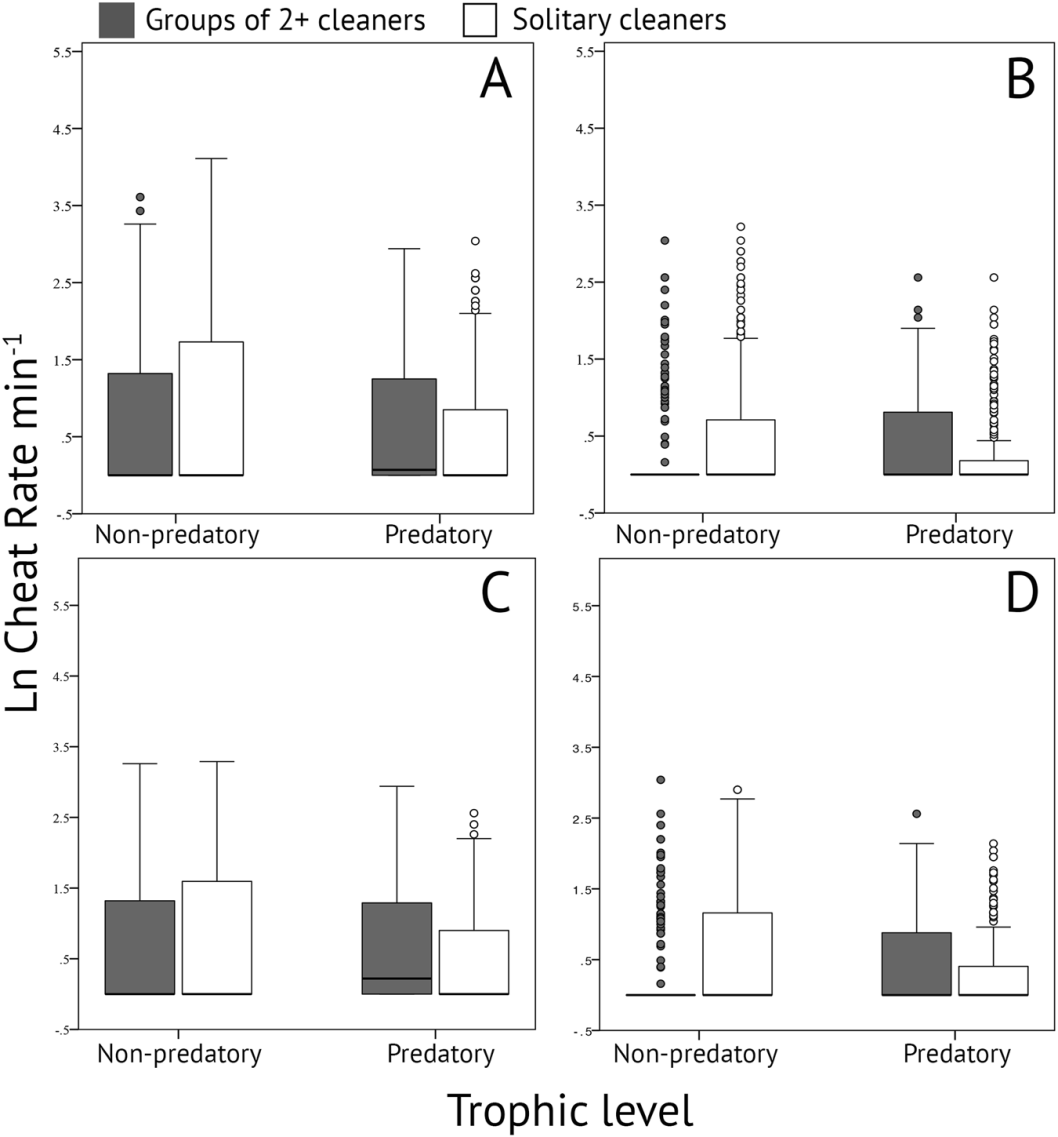
Variation in the natural log of cheating rate (min^−1^) by cleaner shrimp group size (solitary vs group) and client trophic position (predatory vs non-predatory) on client reef fish on coral reefs in the Gulf of Honduras. (**A**) Full dataset, (**B**) Cheating occurrences in the last 5 seconds of a cleaning interaction removed, (**C**) Cleaning interactions less than 5 seconds in total duration removed, and (**D**) Cheating occurrences in the last 5 seconds and cleaning interactions less than 5 seconds in total duration removed. Data are shown as box plots with median and interquartile ranges across cleaner shrimp group size and client trophic position.

When all data were considered (i.e. no cleaning interaction data removed) Generalized Estimating Equations (GEE) demonstrate that cleaner shrimp group size, client trophic position, reef site, and time of day had no statistically significant main effect on the cheating frequency of *A. pedersoni* (Table 2; Fig. 1A). Although the main effect on client trophic position had a p-value of 0.1 and the effect was in the predicted direction (i.e. more cheats on non-predatory clients), groups of cleaners cheated more frequently on predatory clients than non-predatory ones (Fig. 1B). Accounting for potential flinches or jolts from client reef fish that may be signaling the end of their clean, we removed all incidents of putative cheating from the dataset that occurred in the last 5 seconds of the interaction, leaving a final dataset with 623 occurrences of cheating. Again, GEEs demonstrate that cleaner shrimp group size, client trophic position, reef site, or time of day, did not have a statistically significant main effect on cheating frequency (Table 2; Fig. 1B). Eliminating short cleans (≤5 s) resulted in a dataset with 863 cleaning interactions and 799 occurrences of cheating, and there was no statistically significant main effect on cheating rate for cleaner shrimp group size, client trophic position, reef site, or time of day (Table 2; Fig. 1C). Finally, eliminating flinches at the ends of cleans as well as short cleaning interactions length resulted in a dataset with 863 cleaning interactions and 610 incidents of cheating. Again, GEE results show no main effect on cheating rate across the dataset (Table 2; Fig. 1D). There were a handful of significant interaction effects across each GEE (Table 2). For example, for our full dataset, we see a significant interaction effect between trophic level and reef site, which may reflect the importance of local processes on cheating rate (Table 2). We also see significant interaction effects between cleaner shrimp group size and client trophic position in two of our four datasets (Table 2; Fig. 1B,D). Interestingly, in both of these datasets, groups of cleaners appear to cheat more frequently on predatory than non-predatory clients (Fig. 1B,D), which is the opposite of the predicted direction. No interaction effect was significant across all datasets.

**Table 2:** Results of full-factorial Generalized Estimating Equations (GEE) testing for main effects (trophic level, group size, reef site, and time of day) and interaction effects on cheating rate (cheats min^-1^) during cleaning interactions between Pederson’s cleaner shrimp *Ancylomenes pedersoni* and client reef fish on coral reefs in the Gulf of Honduras.

Source	GEE1			GEE2			GEE3			GEE4		
	*χ* ^2^	*df*	*p*	*χ* ^2^	*df*	*p*	*χ* ^2^	*df*	*p*	*χ* ^2^	*df*	*p*
Trophic level (TL)	2.75	1	0.10	0.54	1	0.46	2.39	1	0.12	0.01	1	0.93
Group Size (GS)	0.01	1	0.94	0.13	1	0.72	0.17	1	0.68	0.59	1	0.44
Reef Site (RS)	2.26	2	0.32	0.23	2	0.89	1.00	2	0.61	0.35	2	0.84
Time of Day (TOD)	0.15	2	0.93	0.02	2	0.99	0.54	2	0.76	0.15	2	0.93
TL*GS	1.92	1	0.17	6.46	1	**0.01**	2.79	1	0.10	7.92	1	**0.01**
TL*RS	6.97	2	**0.03**	2.91	2	0.23	9.43	2	**0.01**	3.61	2	0.16
TL*TOD	1.65	2	0.44	3.17	2	0.21	2.84	2	0.24	2.95	2	0.22
GS*RS	3.63	2	0.16	1.58	2	0.45	3.86	2	0.14	2.17	2	0.34
GS*TOD	2.55	2	0.29	12.4	2	**0.00**	5.61	2	0.06	9.95	2	**0.01**
RS*TOD	2.28	3	0.51	17.9	3	**0.00**	2.90	3	0.41	16.4	3	**0.00**
TL*GS*RS	5.23	2	0.07	3.22	2	0.20	4.51	2	0.11	2.75	2	0.25
TL*GS*TOD	0.74	2	0.69	0.89	2	0.64	0.14	2	0.94	1.30	2	0.52
TL*RS*TOD	3.84	3	0.28	4.67	3	0.20	4.61	3	0.20	7.82	3	0.05
GS*RS*TOD	3.48	3	0.32	7.37	3	0.06	12.4	3	**0.01**	8.38	3	**0.04**
TL*GS*RS*TOD	8.16	3	**0.04**	2.58	3	0.46	14.8	3	**0.00**	3.31	3	0.35

GEE1-4 represent analyses conducted on four datasets: GEE1) full dataset, GEE2) incidents of cheating that occurred in the last 5 seconds of a cleaning interaction removed, GEE3) cleaning interactions <5 seconds in total duration removed, and GEE4) cheating incidents that occurred in the last 5 seconds of a clean, and cleaning interactions <5 seconds in total duration removed removed (see Methods).

## Discussion

We demonstrate that Pederson’s cleaner shrimp, *Ancylomenes pedersoni*, do not vary their service quality in the Gulf of Honduras based on cleaner group size, the trophic position of the client reef fish, reef site, or time of day in the hypothesized directions (i.e. more cheating on non-predatory than predatory fish, and more cheating by solitary shrimp than groups of cleaners). Some significant interaction effects were recovered between group size and client trophic position while attempting to account for biologically realistic behaviors in two of our datasets. These effects suggest that the shrimp may be able to discriminate and respond to some aspects of the interaction. What might be causing these remains unclear, and while we cannot rule out some effects of cleaner shrimp group size or client type on cheating rate, the patterns we have recovered are not consistent with our predictions. Additionally, clients of *A. pedersoni* did not leave immediately following a putative cheating event, and did not display any aggressive behavior towards cleaners as has been seen in other studies of this cleaner shrimp.

In light of our results, when considered in conjunction with studies on cleaner gobies^19–21^, it appears that the cleaning system in the Caribbean (cleaners and clients), although functionally and ecologically analogous to those in the Indo-Pacific, have not evolved the same strategic service behaviors or partner control mechanisms. Soares *et al*.^19^ previously noted differences in strategic service behaviors and partner control mechanisms between Caribbean cleaner gobies and *L. dimidiatus*, and we come to similar conclusions regarding the differences in strategic service provisioning between *A. pedersoni* in the Caribbean and *A. longicarpus* in the Indo-Pacific. Soares *et al*.^19^ presented three non-mutually exclusive hypotheses to explain the differences in the evolution of behavioral strategies between cleaner gobies in the Caribbean and cleaner wrasse in the Indo-Pacific that, considering the implications of our findings, may also apply to regional differences between *Ancylomenes* spp. These are (1) the constraint hypothesis, (2) the low cost of cheating hypothesis, and (3) the foraging preference hypothesis.

The constraint hypothesis presents two potential constraints that may explain why Caribbean cleaner systems have not evolved the behavioral complexity of Indo-Pacific systems: (i) Caribbean cleaners are less dependent on a cleaning lifestyle than Indo-Pacific cleaners. Caribbean cleaner gobies, although considered a dedicated cleaner species, have been shown to gain up to 85% of their dietary needs through cleaning while the cleaner wrasse *L. dimidiatus* acquires 99% of their diet through cleaning. Comparable data for cleaner shrimps *A. pedersoni* and *A. longicarpus* do not exist, which are needed to evaluate this hypothesis more broadly. (ii) Differences in cleaning strategy and partner control between ocean basins could arise if cleaners are constrained in their cognitive abilities to remember the interaction and the identity of the client fish that inflicts punishment for cheating. Interestingly, unlike our data on *A. pedersoni*, Chapuis and Bshary^10^ show that *A. longicarpus* can discriminate between predatory and non-predatory clients, although they have no specific hypotheses to explain this ability. Recent research, however, suggests that differentiating between clients visually would not be likely, as two cleaner shrimps from the family Palaemonidae (including *A. pedersoni*) have been shown to have poor spectral sensitivity (i.e. monochromatic vision), and low spatial resolution^32^. Caves *et al*.^32^ conclude that cleaner shrimp vision is sufficient to detect large stimuli, but likely cannot detect sharp outlines, color, or patterns of either potential clients or of conspecific shrimp inhabiting the same cleaning station. Wicksten^33^ suggests that chemosensory setae on shrimp antennae and dactyls could aid in client recognition, and that compounds found in fish mucus could have a pheromonal function^34^. Regardless, the findings from Chapuis and Bshary^10^ demonstrate that complex cognitive abilities can evolve in a closely related shrimp species. Cognitive comparisons between shrimp and fish species within and between ocean basins would be needed to properly test the cognitive constraint hypothesis.

The low cost of cheating hypothesis states that variation exists in the level of cost that a cleaner inflicts on a client when it cheats and removes scales or live tissue rather than parasites. If the cost of being cheated is great enough, selection should drive client strategies to enforce honest behaviors. Soares *et al*.^19^ note that the size differences between the bluestreak cleaner wrasse *L. dimidiatus* (12 cm total length) vs Caribbean cleaner gobies (4 cm total length) may be great enough so that cheating by *L. dimidiatus* could inflict greater injuries on client fish, and thus, inflict a greater cost of cheating. This is yet to be measured, but comparative data from *A. longicarpus* should falsify this hypothesis in terms of its ability to explain differences between ocean basins. Cheating by *A. longicarpus*, a small and delicate palaemonid shrimp certainly cannot inflict the same types of cost on clients as the vertebrate jaws of Caribbean cleaner gobies.

Finally, the foraging preference hypothesis states that differences in the behavioral complexity in cleaning symbioses between ocean basins could be driven by the food preference of the cleaner service providers. Here, the differences between Caribbean and Indo-Pacific cleaner fishes are easy to explain: Caribbean cleaner gobies prefer ectoparasites to client mucus whereas Indo-Pacific cleaner wrasse prefer client mucus to ectoparasites^17,21^. Thus, an inherent incentive to cheat exists for cleaner wrasse but does not for Caribbean cleaner gobies. The differences in food preference between clients means that all interactions with cleaner gobies should begin cooperatively with gobies foraging for parasites. Gobies would only resort to eating scales or fish tissue if parasite abundance is low. The explanation for the behavioral differences between the shrimps *A. pedersoni* in the Caribbean and *A. longicarpus* in the Indo-Pacific is less clear. The foraging preference hypothesis presents the simplest and most consistent explanation for the evolution of different behavioral cleaning strategies in both ocean basins. To our knowledge no studies have evaluated food preference in cleaner shrimp, nor conducted a gut content analysis of *A. pedersoni*.

To summarize, research focused on cleaner shrimp food preference, gut content analyses, and the strategic behaviors of other cleaner species in the Caribbean and Indo-Pacific are needed, but at face value, it appears that cleaning strategies have evolved independently in both ocean basins and that the stability of these mutualisms is maintained in different ways. Advanced signaling, cheating behaviors, body and chelae morphology, hosting with sea anemones, and conspicuous dorsal patterning and coloration have all been noted as convergent properties in species of the genus *Ancylomenes* that have evolved dedicated cleaning lifestyles independently on both Indo-Pacific and Western Atlantic coral reefs^31^. Our data demonstrate that the behaviors that maintain these symbioses are decidedly different.

Although on the whole the Caribbean appears to be a system where the two most prominent cleaner species do not vary their service qualities and clients do not aggressively punish cheating, there are some important differences in the responses of clients to incidents of cheating by gobies and by *A. pedersoni*. Soares *et al*.^19^ studied the frequency of cheating in cleaner gobies in Barbados and found that cheating occurred in ~40% of all cleaning interactions, a value similar to our findings. However, more than 90% of all incidents of cheating by cleaner gobies resulted in the immediate termination of the interaction by the client^19^. Our data show that incidents of cheating by *A. pedersoni* rarely resulted in the client terminating a clean. Even making the most liberal interpretation of our data, only ~27% of all recorded jolts could have resulted in the termination of the interaction. In total, we recorded 163 cleaning interactions with at least 2 incidents of cheating, and 72 cleaning interactions with 3 or more incidents of cheating. Our data clearly demonstrate that clients repeatedly tolerate cheating (or behaviors that resemble cheating) by *A. pedersoni*. While the “low cost of cheating” hypothesis appears to fail to explain differences between ocean basins, it may provide a valid explanation for why clients tolerate cheating more at *A. pedersoni* stations than cleaner goby stations.

An additional hypothesis for increased tolerance to cheating by *A. pedersoni* than by cleaner gobies is their provision of default “massages” during cleaning interactions^10^. Tactile stimulation has been shown to be important for the cleaner wrasse *L. dimidiatus* in manipulating clients into prolonging cleans, reconciling with clients after cheating, and as a pre-conflict strategy towards predators (e.g.^35,36^), but cleaner shrimps may provide tactile stimulation inadvertently during an interaction^10^. Cleaner shrimps are in physical contact with their clients at all times, and shrimps walking across human hands provides a tickling sensation (e.g.^10^; pers. obs.). Providing a similar sensation to clients could then prolong cleans. Further, it may be possible that tactile stimulation may make clients less bothered by cheats, or that these cheats are less noticeable because of the physical sensation of contact with cleaner shrimp. Cleaning interactions at *A. pedersoni* stations have been previously shown to be longer than those at cleaner goby stations, even when servicing the same clients (e.g.^26^). Default massages may reduce stress hormones in clients (e.g.^37^) providing alternative fitness benefits that may outweigh the cost of being cheated, and could partially explain why we see comparatively fewer clients terminate cleaning interactions immediately following a cheating occurrence at shrimp stations.

One aspect of understanding the evolution of strategic service provisioning in cleaner shrimps that deserves more attention is the meaning of client fish jolts and whether it can be confirmed to be a true proxy of cheating. While jolts are largely accepted as a proxy for cheating in the cleaning literature, these studies have come exclusively from cleaner fish [e.g.^6,20^]. In their study of *A. longicarpus*, Chapuis and Bshary^10^ note that they cannot exclude the possibility that fish jolts in response to cleaning by shrimp do not correlate with the removal of live tissue or fish mucus, and that client jolts during cleaner shrimp interactions generated fewer responses than jolts caused by cleaner wrasse. A recent study by Vaughan *et al*.^38^ further casts doubt that the correlation between jolts and cheating is as strong in cleaner shrimps as it is in cleaner fish. Using the Indo-Pacific cleaner shrimp *Lysmata amboinensis* and the reef fish *Pseudanthias squammipinnis* in a manipulative laboratory experiment, Vaughan *et al*.^38^ showed that individual fish clients were responsible for 20x the variance in jolt rate than cleaner shrimp. Fish were moved to different aquaria for each treatment, and the same cleaner-client combination was never re-used. They found that only 10 of 54 *P. squammipinnis* individuals displayed repeated jolting and aggressive behavior towards cleaner shrimp. Although the study by Vaughan *et al*.^38^ uses a different cleaner shrimp species in a non-natural setting, along with only one client species, it’s possible that individual client fish sensitivities are partially responsible for the jolting behaviors we have observed in our study. A targeted study analogous to those that have been conducted with cleaner fish is needed to determine whether non-parasitized clients jolt more frequently in the presence of cleaner shrimps than parasitized clients.

In conclusion, our data present the first evidence that *A. pedersoni*, one of the most ecologically important cleaner species on Caribbean coral reefs, displays no evidence of strategic service behaviors. Because our study is directly comparable to those conducted for cleaner fishes and cleaner shrimp, our work enhances our knowledge of the stability of mutualisms and cleaning behavior on coral reefs, and demonstrates that ecologically analogous cleaning systems in separate ocean basins have evolved independent strategies to regulate and govern these complex behavioral interactions. Like prior research on Caribbean cleaner gobies, our study demonstrates that the cleaning symbiosis involving *A. pedersoni* do not meet all of the expectations set forth by game theoretical models. The fundamentally different ways that cleaning symbioses in the Caribbean have evolved have important implications for future research testing game theoretical models using cleaning systems. Namely, that there is no “standard” strategic service behavior common across cleaning mutualisms. Dozens of fish and crustacean species are classified as cleaner species. Each may have evolved unique behaviors that ultimately result in ecologically important parasite removal from reef fishes. Our understanding of these systems stands to benefit from future studies that explore cleaner shrimp food preference, the cognitive abilities of cleaner shrimps in both ocean basins, and the hormonal responses of client reef fish during cleaning interactions with cleaner shrimps.

## Methods

### Study sites

This study was conducted at three coral reef sites in the Gulf of Honduras at the southern end of the Mesoamerican Barrier Reef: (1) Coral View reef (16°05′ 17.87″N 86°54′ 38.56″W) on the island of Utila (henceforth “Utila”), is a fringing reef on the southern coast of the island that slopes rapidly from ca. 3–30 m depth, (2) an unnamed reef (15°57′ 00.55″ N 86°29′ 49.19″ W) in the Cayos Cochinos Marine Protected Area (henceforth “Cayos Cochinos) is a fringing reef sloping from ca. 1.5–20 m, and (3) a newly discovered reef, Banco Capiro, in the Bay of Tela (15°51′48.71″N, 87°29′42.90″W), is an offshore reef 8 km from the mainland ranging from a crest at 10 m depth and sloping to beyond 30 m. Both Utila and Cayos Cochinos reefs are typical contemporary Caribbean reefs, with oligotrophic nutrient conditions, similar percent live coral cover, reef fish abundance, and reef fish community structure (detailed by^25,26^). Banco Capiro, however, is characterized by unusually high percent live coral cover for a contemporary Caribbean reef (~49–65%), meso- to eutrophic nutrient loads given its close proximity to the mainland, and a reef structure comprised largely of flattened, plating, *Orbicella annularis*, a coral growth form typically found at greater depths (>30 m^39^). Each reef site is ~45–60 km from each other. Although we are primarily focused on the strategic behaviors exhibited during individual cleaning interactions, the ecological reef setting is known to drive variation in ectoparasite abundance, client parasite loads, and the rate at which clients seek cleaners. How, or whether, the ecological setting generates variation in strategic service behaviors in cleaner shrimps is unknown. Greater ecological detail on each reef site can be found elsewhere^25,26,39^.

### Behavioral observations

At each reef site, corkscrew sea anemones, *Bartholomea annulata*, that hosted *A. pedersoni* cleaner shrimps were tagged (to avoid duplication), measured (tentacle crown surface area cm^2^; see^24,40^), and mapped. Finally, the number of *A. pedersoni* shrimp in each were recorded. While *A. pedersoni* will host with other species, *B. annulata* is overwhelmingly the most common sea anemone host for this species^41^. The symbiosis with *B. annulata* forms the hub of these mutualistic cleaning interactions between shrimp and client reef fish, and the anemones are thought to serve as visual cues for reef fish locating cleaning stations^24^.

To reduce the effects of diver presence on reef fish behavior (e.g.^25^) and provide a greater overall time of observation than direct SCUBA diver observations, behavioral observations at each cleaning station were recorded using remotely deployed GoPro underwater cameras. Following Titus *et al*.^25–28^, cameras were deployed remotely by SCUBA divers to randomly assigned *A. pedersoni* cleaning stations. Cameras were attached to dive weights and deployed 1–1.5 m from each cleaning station, and allowed to record continuously for the duration of the battery life (120–180 minutes). SCUBA divers immediately exited the water following camera deployment. Although time of day has been shown to have no effect on shrimp cleaning rate on reefs in the Bay Islands^26^, the time of day of camera deployment was recorded (e.g. AM, midday, PM) and accounted for in statistical analyses.

Videos were later analyzed for: client reef fish identity (to species level), client trophic position (predatory vs non-predatory), cleaner shrimp group size (solitary vs groups ≥ 2), interaction length (s), cheating frequency, time of day (AM, midday, PM), and reef site (Utila, Cayos Cochinos, and Tela). Following Chapuis & Bshary^10^, we classified cleaner shrimp clients into predatory and non-predatory trophic positions based on diet. Diet information was obtained from FishBase^42^. A cleaning interaction was defined as contact with a client fish’s body (however brief) by the cleaner shrimp (e.g.^23–28^). Cheating frequency was quantified by counting the number of client “jolts”, or “flinches”, during the cleaning interactions, shown previously to be an accurate proxy for cheating frequency^18,20^. The number of cheating events were standardized by calculating a cheating rate (jolts/flinches min^−1^) to account for variation in the length of each cleaning interaction. In some videos, potential client reef fish posed at the anemone but were not cleaned by the shrimp; these visits were not included in the final analysis as there was no physical engagement between cleaner and client. Additionally, during some interactions, cleaner gobies from adjacent cleaning stations were present and participated in the cleaning process simultaneously. Cleaning interactions where both cleaner species were present were omitted from the dataset as we were unable to distinguish whether cleaner shrimp or cleaner fish were responsible for any cheating that occurred.

All fieldwork was observational, non-extractive data collected, conducted under permit number 19985 issued by the Honduran government.

### Statistical analysis

Cheating rate data did not conform to a normal distribution and so it was natural log (ln) transformed to be analyzed statistically using Generalized Estimating Equations (GEE) with a normal distribution and identity link functions. Because multiple cleaning interactions occurred at the same anemone cleaning stations, station identity was used as a repeated measure subject effect. Each GEE was conducted with a maximum likelihood scale parameter and Bonferroni correction for multiple pairwise comparisons. We tested for main effects on cheating rate for the following fixed factors: client trophic position, cleaner group size, time of day, and reef site. For each GEE we conducted a full factorial analyses to explore possible interaction effects between each fixed factor.

To ensure the robustness of our data statistically, account for biologically realistic cleaning interaction behaviors, and ensure we are being conservative in our conclusions, we conducted four separate GEEs to understand the effect of client trophic position and cleaner shrimp group size on service quality: (1) all data collected throughout the entirety of each cleaning interaction, (2) removal of cheating occurrences from the data set that occurred during the last five seconds of a cleaning interaction where jolts or flinches by client fish could also be interpreted to be a signal by the client to terminate the clean (Supplementary Video 1), (3) removal of brief cleaning interactions (≤5) from the dataset due to the potential of these exceedingly short cleans to bias our results, and (4) removal of both cheating occurrences in the last five seconds of an interaction and the removal of brief cleaning interactions. The rationale behind removing short cleaning interactions is that many of these brief cleans had no cheating occurrences and may have been too short to discern any meaningful behaviors.

## Supplementary information

Supplementary Video 1

Supplementary Dataset 1

## Data Availability

All data from which analyses were performed are available as Supplementary Data.
